# Are Saudi Healthcare Workers Willing to Receive the Monkeypox Virus Vaccine? Evidence from a Descriptive-Baseline Survey

**DOI:** 10.3390/tropicalmed8080396

**Published:** 2023-08-02

**Authors:** Abdullah M. Alarifi, Najim Z. Alshahrani, Ranjit Sah

**Affiliations:** 1 Department of Public Health, College of Health Sciences, Saudi Electronic University, Riyadh 13323, Saudi Arabia; 2 Department of Family and Community Medicine, Faculty of Medicine, University of Jeddah, Jeddah 21589, Saudi Arabia; 3 Department of Microbiology, Tribhuvan University Teaching Hospital, Institute of Medicine, Kathmandu 44600, Nepal; 4 Department of Microbiology, Dr. D. Y. Patil Medical College, Hospital and Research Centre, Dr. D. Y. Patil Vidyapeeth, Pune 411018, India; 5 Department of Public Health Dentistry, Dr. D.Y. Patil Dental College and Hospital, Dr. D.Y. Patil Vidyapeeth, Pune 411018, India

**Keywords:** Monkeypox vaccine, vaccine willingness, Mpox, healthcare workers, Saudi Arabia

## Abstract

Since Saudi Arabia has already confirmed multiple monkeypox (Mpox) cases, it is essential to initiate timely preventive measures, including the implementation of vaccines. In this cross-sectional study, an online survey was conducted among healthcare workers (HCWs) in Saudi Arabia to understand their willingness to receive the Mpox vaccine. A structured questionnaire was used to gather the data. The study comprised 734 samples. Our study found that among study participants, 52.7% were willing to receive the Mpox vaccine and showed that sociodemographic factors were not significantly associated with vaccine willingness. Previous vaccination history (such as influenza and COVID-19) was significantly associated with Mpox vaccine willingness. The respondents reported that the main reasons for receiving the Mpox vaccine were their trust in the Saudi Health Ministry (57.7%) and their understanding that the vaccine was a social responsibility (44.6%). Furthermore, the majority of the respondents (74.7%) reported that they were motivated by the need to protect themselves, their family and their friends. Insufficient vaccine information and fear of unknown adverse reactions were the most reported reasons for an unwillingness to receive the Mpox vaccine. In conclusion, increasing Mpox vaccine-related awareness and focusing on greater information dissemination to reduce fear and increase vaccine uptake is highly recommended.

## 1. Introduction

The world has experienced the ways in which coronavirus (COVID-19) affected people’s quality of life, as well as physical and mental health and well-being [1,2,3]. By the autumn of 2022, COVID-19 had affected more than 630 million people worldwide, causing more than 6.5 million deaths [4]. Recently, the World Health Organisation (WHO) announced that COVID-19 was no longer a “global health emergency”, but also emphasised that it was still a threat to global health [5]. Currently, different regions in the world are facing another emerging infectious disease known as monkeypox (Mpox) [6]. The WHO has declared this a global public health emergency [7,8]. There have already been close to 80,000 confirmed cases of Mpox globally, and Mpox cases have been reported in 110 nations, with 50 confirmed deaths [9]. Although these figures do not sound massive, experts have warned against the potential risk of underestimating the threat or responding too late. Studies regarding the COVID-19 outbreak show that the lack of vaccine availability in the early stages of the epidemic and improper disease containment strategies were behind the spread of infection. Hence, experts warn that if similar mistakes are repeated with Mpox, it may become a more significant threat [10].

Evidence shows that vaccine hesitancy and refusal are complex phenomena, leading to serious health risks [11,12]. For instance, there are several COVID-19 vaccines approved for use, but many people remain unvaccinated. It is for this reason that researchers refer to vaccine hesitancy as the most significant health threat and as a driving force behind the pandemic [13,14]. This only underlines the importance of identifying the factors influencing the willingness to receive the vaccine in different population groups. In the case of Mpox, vaccines are already available, since research suggests that smallpox vaccines are highly effective in countering infection [15,16,17,18,19,20]. Thus, the JYNNEOS vaccine and ACAM2000 vaccine can help in preventing the spread of infection [21,22]. Moreover, several studies used computational approaches to design a multi-epitope vaccine against Mpox [23,24].

Mpox disease is caused by the monkeypox virus, which is a double-stranded DNA virus belonging to the *Orthopoxvirus* genus and Poxviridae family [9]. This virus has two different genetic clades: Clade I and Clade II. Mpox causes a self-limiting illness lasting 2–4 weeks, and has a mortality rate of 3–6%. It is transmitted by coming into close contact with a person or animal infected with the virus. Its clinical picture somewhat resembles smallpox, with widespread lesions. In this condition, skin eruptions begin 1–3 days after the appearance of fever [25]. Lesions are commonly present on the face, hands and soles of the feet, mucous membranes, and genitalia. The infection is mainly endemic to West Africa, in nations like the Democratic Republic of the Congo, Nigeria, Gabon, Sierra Leone and Cote d’Ivoire. However, outbreaks outside Africa have previously been reported, like the 2003 outbreak in the US [21]. Nonetheless, the current outbreak is unique, as it is the first time that Mpox has been reported in so many nations. Although the total number of Mpox cases reported in Saudi Arabia remains low, it still poses a significant public health threat. To date, fewer than 10 Mpox cases have been confirmed in Saudi Arabia [26]. However, it is likely that Mpox cases are under-reported [9]. Most of the Mpox cases in Saudi Arabia have been imported from other countries, which also means that tourism, a huge sector in Saudi Arabia, poses a significant threat [26].

The recent Mpox outbreak also highlighted the need for continuing progress in the development of novel treatment and preventive techniques to combat infectious disease threats [27,28]. The availability of Mpox vaccines does not ensure their acceptance among the most at-risk populations, which include healthcare workers (HCWs), patients with immunodeficiencies and men who have sex with other men (MSM) [29,30,31,32,33]. For example, a study showed a low intention to get Mpox vaccination among HCWs (such as nurses and physicians) in Jordan [34]. Another study conducted among Ghanaians reported that they did not show high levels of intention to receive the Mpox vaccination [35].

Vaccination campaigns must be initiated with HCWs since they are those most likely to come in contact with the infection. Early experience with COVID-19 shows a lack of understanding of the risk posed by infection on the part of travellers, as well as vaccine hesitancy exacerbating the epidemic in Saudi Arabia [18]. Moreover, educating the population about the risks requires effort, as shown by the lack of awareness and slow uptake of the COVID-19 booster dose in Saudi Arabia [17]. In terms of Mpox, recent studies have reported that there is a lack of sufficient knowledge about the Mpox disease among the general population and HCWs in Saudi Arabia [36,37,38,39]. Apart from the global spread of Mpox and vaccine hesitancy, other significant causes for concern in Saudi Arabia include tourism within the nation and neighbouring countries and major events like the Hajj and Omrah [26]. Similarly, another threat is posed by people visiting the Gulf nations, especially from South Asia. Data show that the UAE and Saudi Arabia are among the most common destinations for South Asians and the identification of Mpox in these nations cannot be neglected [22]. Hence, there is a need to introduce Mpox vaccination to HCWs in Saudi Arabia. Additionally, based on early experience, understanding the willingness to receive the Mpox vaccine and identifying the factors affecting vaccine acceptability are the need of the hour. Thus, this descriptive study was designed based on the following three objectives: (i) to assess the willingness of HCWs to receive the Mpox vaccine in Saudi Arabia; (ii) to investigate reasons and motivating factors for receiving the Mpox vaccine; and (iii) to identify the reasons for unwillingness to receive the Mpox vaccine.

## 2. Materials and Methods

### 2.1. Study Design and Participants

This was an online-based cross-sectional study, which was conducted among HCWs including physicians, nurses, pharmacists, etc., in different regions of the Kingdom of Saudi Arabia (i.e., the northern, western, middle, southern and eastern regions) from 13 September 2022 to 13 November 2022. HCWs from primary healthcare, secondary healthcare, tertiary healthcare (hospitals) and private clinics from all regions in Saudi Arabia aged >20 years were invited to participate in the survey. The study excluded participants who did not complete the survey questionnaires. Additionally, individuals who gave delayed responses were not included in the study. 

### 2.2. Sample Size Estimation 

The sample size was calculated based on the single sample proportion test. The formula is as follows: n = Z^2^ × P × (1 − P)/e^2^. Here, n = the minimum number of samples required; Z = 95% confidence interval (1.96); P = predicted prevalence of the outcome. Since no comparable study has been conducted in Saudi Arabia, we assumed that 50% of HCWs would be willing to receive the Mpox vaccine (i.e., *p* = 0.5), and e = margin of error of 4%. Thus, a minimum sample size of 600 participants was calculated for this study. 

### 2.3. Ethical Approval

The study was ethically approved by the committee of medical ethics at the Security Forces Hospital Program in Makkah (HAP-0 2-K-052). Consent was obtained from all participants at the beginning of the survey after conveying the study’s objective, research method and their rights. Participants’ confidentiality was protected at all times of the study. In addition, the study followed the ethical guidelines set forth by the Declaration of Helsinki.

### 2.4. Data Collection Procedures

Using a Google Survey Link, the online questionnaire was distributed to the random HCWs. Online platforms such as Telegram, WhatsApp and Email were used to disseminate the survey links. The questionnaire was pre-structured and piloted among 15 HCWs to understand the contents. The content of the questionnaire was clear and flexible during the pre-test. The pre-tested data were excluded from the final study. Each response took approximately 10–12 min to complete. Excel was used to store the data for further statistical analysis.

### 2.5. Questionnaire Survey

An anonymous self-report survey was applied to investigate respondents’ attitudes and acceptance of the Mpox vaccines. The survey questionnaire was retrieved from the previous studies performed in China and Indonesia and was therefore regarded as having been validated [40,41]. It was written in English and divided into three main sections: (i) demographic data and previous vaccination history, which includes the participant’s age, gender, marital status, nationality, and education level; and (ii) the participant’s attitude towards Mpox vaccines. To assess the participant’s acceptance, the following questions were asked: “Do you think Mpox vaccination will reduce the risk of Mpox infection and its complications?” “Do you think vaccination is necessary to control Mpox infection?” “Are you worried about the possible side effects of Mpox vaccines?” “Are you concerned about the effectiveness of Mpox vaccines?” “Are you concerned about the safety of Mpox vaccines?” “Are you worried about the defects of Mpox vaccines?” “Are you willing to receive the Mpox vaccine only with enough information available?” “Do you think Mpox vaccination should be made compulsory?” and “Would you encourage your parents and friends to receive Mpox vaccination?”; (iii) it was concluded with the survey to measure participants’ willingness to accept Mpox vaccination with the choices of “yes” and “no”. Out of all of the participants, those who chose yes were regarded as belonging to the “vaccine acceptance” group. On the other hand, those who chose no were thought to belong to the “vaccine hesitancy” group. Additionally, the survey also included infodemic-related questions such as what sources of information the respondents tested, like the Health Ministry of Health in Saudi Arabia (MOH), the Centers for Disease Control and Prevention (CDC), religious leaders, and others.

### 2.6. Statistical Analysis 

The statistical analysis was performed using SPSS version 21.0 (Chicago, IL, USA). Numbers and percentages were used to convey participants’ demographic characteristics and responses. The chi-square test was used to evaluate the relationships between independent variables (demographical and sociological characteristics in addition to their attitudes toward Mpox vaccines) and dependent variables (participants’ willingness to receive the Mpox vaccines). *p* < 0.05 was considered statistically significant. 

## 3. Results

### 3.1. Sample Characteristics 

The invitation was sent to 800 HCWs; of these, 23 did not respond, or refused to participate in the study, while another 34 did not complete the survey. Thus, 743 responses were included in the study. This means that the survey had a response rate of 92.9%. The participation of males and females was almost equal. More than two-thirds (68%) of the respondents were Saudi nationals and the rest of them were non-Saudi. Overall, 40% of the respondents were physicians, 28.5% were nurses, and the rest were pharmacists and other HCWs. 

### 3.2. Willingness to Receive an Mpox Vaccine

Out of the respondents, 392 (52.7%) were willing to receive the Mpox vaccine and 351 (47.3%) were not. It appears that the age of HCWs did not influence the willingness to receive the vaccine, with close to 50% being willing to receive the vaccine in various age groups. However, those older than 56 years were more willing (61.9%). Similarly, gender, marital status and nationality did not significantly influence the willingness to get vaccinated. However, those with lower education, that is, below diploma level, were more willing (58.1%) than those with a PhD or equivalent (39.7%) (Table 1).

### 3.3. Professional Characteristics and Previous Vaccine Behaviour of HCWs and Willingness to Receive the Mpox Vaccine

The study found that physicians and pharmacists were more willing to receive the Mpox vaccine (57.5% and 56.1%) than nurses (46.7%). There was also a significant association between vaccine acceptability and professional experience, with those with fewer than 5 years of experience being more willing (53%) than HCWs with experience exceeding 10 years (46.6%). However, when it comes to the nature of the work, those working in hospitals were less likely (44.7%) to opt for the vaccine than primary healthcare workers (57.9%). In addition, 61.6% of the participants who regularly received the influenza vaccine were willing to receive the Mpox vaccine compared to 49.4% who never received the influenza vaccine (*p* < 0.001). There was a significant relationship between previous history of COVID-19 vaccination and willingness to receive the Mpox vaccine (54.1% vs. 33.3%, *p* = 0.005) (Table 1). 

### 3.4. Attitudes to Other Vaccines and Willingness to Receive the Mpox Vaccine

Data show that those who got the influenza vaccine annually were more willing to get vaccinated (61.6%) than those who had never had an influenza shot (49.4%). Those who were concerned about monkeypox infection were more likely to get vaccinated than those who were not concerned (69.2% vs. 43.2%, respectively). If a person knew someone who had become ill due to monkeypox, it significantly increased their willingness to get vaccinated (70.3%). Those who received the COVID-19 vaccine were significantly more likely to opt for the vaccine (54.1%) than those who did not (33.3%).

### 3.5. Reasons for Willingness to Receive the Vaccine

**Figure 1:**
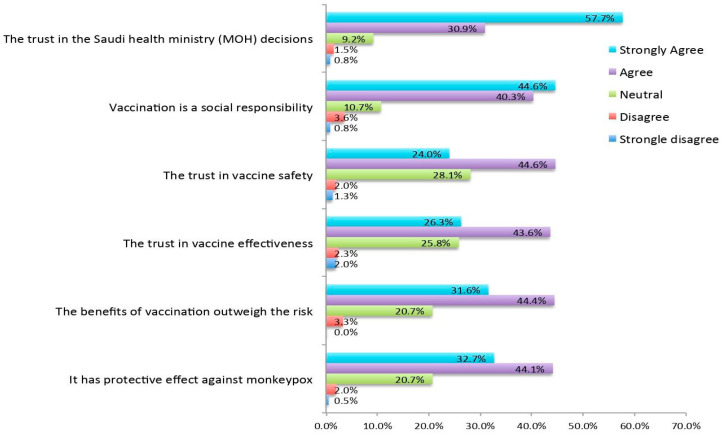
Reasons to receive the Mpox vaccine.

Those who were willing to receive the vaccine said that the main reason is their trust in the Saudi Health Ministry (MOH) (57.7%) and their understanding that the vaccine is a social responsibility (44.6%). Other reasons to get vaccinated were trust in vaccine safety, effectiveness, benefits outweighing risk and confidence that it is protective against monkeypox (Figure 1).

**Figure 2:**
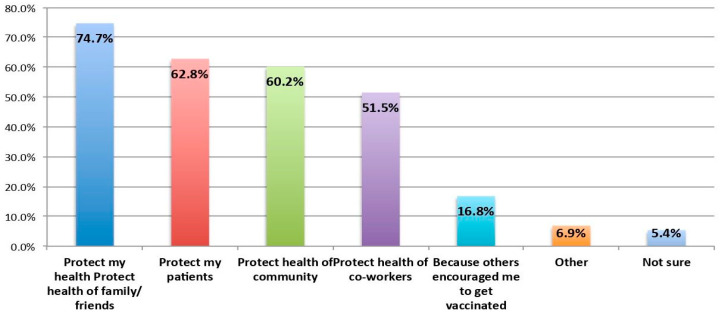
Motivating factors for receiving the Mpox vaccine.

Furthermore, 74.7% of respondents said they were motivated (Figure 2) by the need to protect their health, family and friends. Another 60–62.8% were motivated by the reason to protect patients and the community. Protecting the health of co-workers was also a significant motivating factor.

### 3.6. Reasons Why Unwilling to Receive the Mpox Vaccine

**Figure 3:**
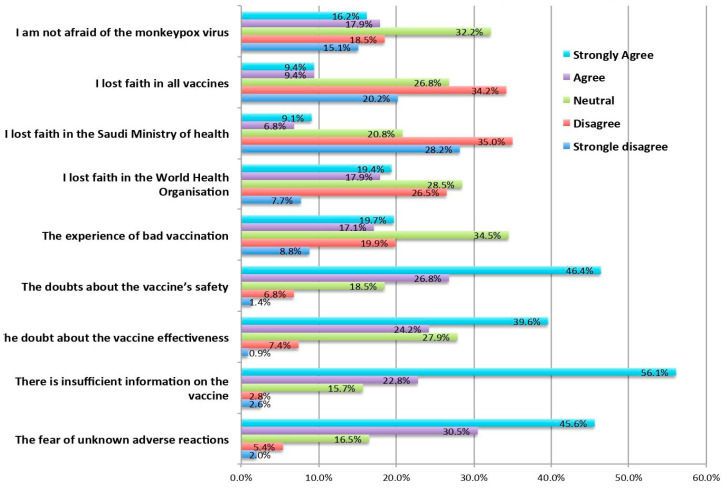
Reasons for unwillingness to receive the Mpox vaccine.

When it comes to identifying the reasons for unwillingness, the most common reason was insufficient vaccine information, fear of unknown adverse reactions, and doubts over vaccine effectiveness and safety (Figure 3).

## 4. Discussion

Mpox has long been endemic to parts of Africa, mainly in western Africa, with sporadic and self-limiting outbreaks outside the continent. However, the present outbreak is different: it is the first major outbreak with cases reported in 110 nations [9]. This has led to the WHO calling it a global public health emergency [8]. There are reasons for increased concerns, such as the virus adapting to the human body and undergoing mutations. Hence, there is a risk that infection may become more widespread [42], and it is essential to stop the spread of the infection in its early stages. Since the world is more interconnected, the only way to prevent global pandemics is by ensuring that each nation takes adequate and timely measures. At present, very few cases have been reported in Saudi Arabia, and these have been imported. However, there are chances of under-reporting. Additionally, there is a significant risk that many more cases might be imported to Saudi Arabia, resulting in human-to-human disease transmission. Since HCWs are at the greatest risk of exposure, any vaccination effort must begin with them. Fortunately, smallpox vaccines are highly effective at providing sufficient immunity against the infection. If such a need arises, understanding the attitudes of HCWs towards the vaccines may help to devise better policies in initiating vaccine campaigns focused on the general population. This is one of the first studies to explore HCWs’ willingness to receive Mpox vaccines in Saudi Arabia. The study had many interesting findings.

In our study, more than half of the respondents (52.7%) were willing to receive the Mpox vaccine. A recent study conducted among Chinese HCWs reported that 36.24% of them expressed a strong willingness to receive the Mpox vaccines and 53.88% were likely to be willing to receive the Mpox vaccines [43]. Moreover, the overall vaccine acceptance rate among Chinese HCWs was 90.12% [43], which is higher than in our study sample. In Jordan, nurses and physicians participated in a study by Mahameed et al. [34], which found that 28.9% of the participants intended to take the Mpox vaccination, 33.3% were hesitant and 37.8% were resistant. Although Mpox vaccine acceptance prevalence among HCWs was higher in our study samples than that of found in Jordan [34], a meta-analysis reported that the pooled prevalence of Mpox vaccine acceptance was 63% among HCWs [44]. Moreover, a recent study by Lounis and Riad raised concerns about potential Mpox vaccination hesitancy among health professionals despite greater rates of vaccine uptake compared to the rates reported among the general population globally [45]. In contrast to our findings, HCWs in Algeria (39%) and the Czech Republic (9.0%) have lower rates of Mpox vaccine acceptance or favour [45,46]. 

Interestingly enough, the study did not find a significant relationship between vaccine willingness and various sociodemographic factors. Similarly, another Chinese study found that most demographic characteristics, such as gender, residence, education level, occupation and department, had no impact on vaccination [43]. Thus, age, gender, marital status and education had a moderate influence. Nonetheless, the study found that older HCWs, that is, those aged 56 years or above, were more willing to receive vaccines, which may be explained by the higher risk of health-related complications in this age group. Interestingly enough, highly educated HCWs with a PhD or above were significantly less willing (39.7%) than those with below-bachelor-level education (58.1%). This could be because there have been a smaller number of reported cases in Saudi Arabia, and thus many highly trained HCWs may not have regarded Mpox as a significant health threat. It is important to note that there is evidence of an association between participants’ Mpox vaccine willingness and their sociodemographic characteristics [47,48,49]. 

Studies have demonstrated that prior vaccination behaviour is a key factor in determining vaccine uptake [17,20,50]. Our study found that early experience with vaccines like influenza and the COVID-19 vaccine had a significant impact on Mpox vaccine willingness. Similarly, a recent study showed that previous vaccination history (including the influenza and COVID-19 vaccines) is a significant predictor of willingness to receive the Mpox vaccine [43]. Another study revealed that participants who refused to receive the COVID-19 vaccine also refused the Mpox vaccine [48]. Thus, initiatives to prevent vaccination resistance or hesitancy may be more effective if they pay particular attention to those who have a history of poor vaccine uptake for various vaccine types. Additionally, our study found that although professional characteristics like a current role as a physician or nurse, income level and administrative or clinical jobs matter, nevertheless, such characteristics have a minimal impact on willingness to receive the vaccine. Interestingly, highly experienced professionals with experience exceeding 10 years were less willing to receive vaccines than less experienced professionals (46.6% vs. 53%). 

Some of the most significant reasons for not getting vaccinated were a lack of sufficient information (56.1% strongly agreed), fear of unknown side effects (45.6% strongly agreed) and doubts about vaccine effectiveness (46.4% strongly agreed and 26.8% agreed). However, more than half of the respondents strongly agreed that they trusted official information from the MOH, which underlines the importance of disseminating vaccine-related information. As expected, HCWs realised that they had a social responsibility to get vaccinated. From the above findings, it is clear that the MOH and other government bodies must be more active in spreading the word and raising awareness about the vaccine. The MOH should also be faster at providing approvals for vaccines, as this would result in greater confidence in vaccines.

### Study Limitations

Although the findings of the study are applicable to HCWs, they are not at all representative of the general population. HCWs are more knowledgeable about the risks posed by specific infectious agents, and they are also aware of the benefits of vaccines. Therefore, additional studies might be needed to understand the general population’s attitude toward the Mpox vaccine. Additionally, it is worth keeping in mind that the study was performed when there were fewer than 10 confirmed Mpox cases in Saudi Arabia. However, if the number of cases increases, the willingness to receive vaccines might change considerably. Finally, self-reporting and social desirability biases may exist in our data. 

## 5. Conclusions

In summary, the present study found that more than half of the respondents were willing to get vaccinated. Previous vaccination history (such as influenza and COVID-19) was significantly associated with Mpox vaccine willingness. The main reason to receive the Mpox vaccine was people’s trust in the Saudi Health Ministry and their understanding that the vaccine was a social responsibility. Furthermore, the majority of respondents reported that they were motivated by the need to protect themselves, their family and their friends. Insufficient vaccine information and fear of unknown adverse reactions were the most reported reasons for the unwillingness to receive the Mpox vaccine. Since this study provides baseline evidence, policymakers can use the outcomes of this study to develop and design initiatives to prevent the Mpox outbreak. Increasing Mpox vaccine-related awareness and focusing on greater information dissemination to reduce fear and increase vaccine uptake is highly recommended. Future analytical studies considering country-representative samples and other influencing factors such as psychological factors are recommended in order to identify the causal factors of Mpox vaccine willingness.

## Figures and Tables

**Table 1:** Relationship between willingness to receive the vaccine and sociodemographic factors.

Characteristic			Willingness to Receive the (Mpox) Vaccine		Total (743)	*p* Value
			No 351 (47.3%)	Yes 392 (52.7%)		
Sociodemographic Characteristics						
Age (in years)	<25	N	173	205	378	0.781
		%	45.8	54.2	100.0	
	26–35	N	121	125	246	
		%	49.2	50.8	100.0	
	36–45	N	32	33	65	
		%	49.2	50.8	100.0	
	46–55	N	17	16	33	
		%	51.5	48.5	100.0	
	>=56	N	8	13	21	
		%	38.1	61.9	100.0	
Gender	Female	N	178	200	378	0.933
		%	47.1	52.9%	100.0	
	Male	N	173	192	365	
		%	47.4	52.6	100.0	
Marital status	Single	N	191	224	415	0.428
		%	46.0	54.0	100.0	
	Married	N	141	154	295	
		%	47.8	52.2	100.0	
	Divorced/Widowed	N	19	14	33	
		%	57.6	42.4	100.0	
Nationality	Non-Saudi	N	111	127	238	0.821
		%	46.6	53.4	100.0	
	Saudi	N	240	265	505	
		%	47.5	52.5	100.0	
Education level	Below diploma	N	26	36	62	0.083
		%	41.9	58.1	100.0	
	Diploma	N	29	28	57	
		%	50.9	49.1	100.0	
	Bachelor’s	N	220	272	492	
		%	44.7	55.3	100.0	
	Master’s	N	41	33	74	
		%	55.4	44.6	100.0	
	PhD or equivalent	N	35	23	58	
		%	60.3	39.7	100.0	
Work Experience	<5 years	N	205	233	438	0.210
		%	46.8	53.2	100.0	
	5 to 10 years	N	83	104	187	
		%	44.4	55.6	100.0	
	>10 years	N	63	55	118	
		%	53.4	46.6	100.0	
Previous Vaccine Behaviour						
Got Influenzas’ vaccine before?	Never got before	N	82	80	162	<0.001 *
		%	50.6	49.4	100.0	
	Yes, in annual intervals	N	117	188	305	
		%	38.4	61.6	100.0	
	Yes, in irregular intervals	N	152	124	276	
		%	55.1	44.9	100.0	
Received a COVID-19 vaccine including booster doses?	No	N	32	16	48	0.005 *
		%	66.7	33.3	100.0	
	Yes	N	319	376	695	
		%	45.9	54.1	100.0	

* Statistically significant (i.e., *p* < 0.05).

## Data Availability

All data that support the findings of this study are available from the corresponding author upon reasonable request.
